# Improving equity and wellness in cancer care with people of Latin American and African Descent: a study protocol

**DOI:** 10.3389/fonc.2025.1469037

**Published:** 2025-02-26

**Authors:** Anna Santos Salas, Sharon M. Watanabe, Aynharan Sinnarajah, Nahyeni Bassah, Fleur Huang, Jacqueline Alcalde-Castro, Harkeert Judge, Hannah M. O'Rourke, Pilar Camargo Plazas, Bukola Salami, María J. Santana, Omar Abdel Rahman, Iqmat Iyiola, Tracy Wildeman, Lisa Vaughn, Sadia Ahmed, The Patient Advisory Council

**Affiliations:** ^1^ Faculty of Nursing, College of Health Sciences, University of Alberta, Third Floor Edmonton Clinic Health Academy, Edmonton, AB, Canada; ^2^ Division of Palliative Care Medicine, Department of Oncology, Faculty of Medicine and Dentistry, College of Health Sciences, University of Alberta, Edmonton, AB, Canada; ^3^ Department of Medicine, Queen’s University, Kingston, ON, Canada; ^4^ Division of Radiation Oncology, Department of Oncology, Faculty of Medicine and Dentistry, College of Health Sciences, University of Alberta, Edmonton, AB, Canada; ^5^ Department of Palliative Care, Princess Margaret Cancer Centre, University Health Network, Toronto, ON, Canada; ^6^ School of Nursing, Queen’s University, Kingston, ON, Canada; ^7^ Cumming School of Medicine, University of Calgary, Calgary, AB, Canada; ^8^ Alberta Strategy for Patient Oriented Research Patient Engagement Unit, University of Calgary, Calgary, AB, Canada; ^9^ Medical Oncology Cross Cancer Institute and Department of Oncology, Faculty of Medicine and Dentistry, College of Health Sciences, University of Alberta, Edmonton, AB, Canada

**Keywords:** cancer, Latin American people, black people, health equity, patient-oriented research, community-based participatory research, palliative care

## Abstract

**Background:**

Cancer inequities such as late access to cancer screening and diagnosis affect people of African and Latin American descent in Canada. These inequities in addition to experiences of racism and discrimination and unequal living and working conditions are detrimental to their wellness. We aim to delineate together with people of African and Latin American descent a patient-oriented pathway to improve their equity and wellness in cancer care.

**Methods:**

This is a 3-year community-based and patient-oriented participatory research study. The study will take place in Alberta and Ontario and will involve 125 participants including people with cancer, family and community members of African and Latin American descent, and health care providers. We will conduct in-depth interviews with patients and families and focus groups with community members. Together with patient partners and community collaborators, we will delineate a patient-oriented pathway in cancer care to improve equity and wellness for people of African and Latin American descent in Canada. Finally, we will explore the acceptability of the pathway with a small sample of patients, families and health care providers.

**Conclusion:**

This study will advance our knowledge of equity and wellness in people with advanced cancer from racialized communities in Canada; and increase our understanding of how racialized populations live through a cancer diagnosis. The study will also generate knowledge of how a patient-oriented health equity pathway can contribute to reduce cancer inequities in the care of our study populations.

## 1 Introduction

In Canada, cancer disparities affecting members of racialized populations and people from low income areas exist (1–6). These disparities are evidenced as lower rates of access to screening, diagnosis, and curative treatments, higher rates of late cancer diagnoses (7), and lower survival rates than the general population (1, 3). Racism, discrimination, lack of racially diverse providers, lack of trust in the health care system, and unequal living and working conditions signal inequities that can be detrimental to the health and wellness of racialized patients with cancer (8–12). Black and Latin American peoples in Canada experience significant socioeconomic inequities including higher rates of unemployment, working poverty, and discrimination (4, 13). Significant cancer disparities affecting these groups elsewhere are known (14–16). In Canada, emerging evidence points to concerning cancer disparities in these population groups (3, 17–19).

The collection of race or ethnicity- based population health data in Canada is limited (20), hindering comprehensive reporting of cancer outcomes for members of racialized communities. A cancer incidence study utilizing population data from 2006 reported that African, Caribbean, and Latin American groups had the highest incidence of prostate cancer among other ethnicities in Canada (19). However, Latin American and Black peoples in Canada may experience suboptimal cancer care outcomes. Low cancer screening rates for Latin American and Black women have been reported in Canada (21, 22). Women from Sub-Saharan Africa and Latin America undergoing breast cancer surgery in Canada were reported to have a higher burden of pain (23). End-of-life cancer care disparities were identified for Latin American and Black peoples in an Ontario study (24). Cancer care for these communities is a health equity concern, as there are unfair and avoidable systemic social conditions affecting their full potential for health and wellbeing (25). There is a need to explore how patients with advanced cancer of African and Latin American descent in Canada experience equity and wellness.

This study will provide an opportunity to learn from their culturally diverse perspectives and explore health equity pathways to support Black and Latin American peoples’ experiences with advanced cancer in Canada. We have adopted a community wellness perspective (26), which pays attention to people’s “personal, relational, and collective” experiences and brings a social justice approach into their care (27)^p.54^. 

A focus on wellness follows a strengths-based approach that redirects attention to who people are, their characteristics and strengths, and the context of their lives, to increase health and wellbeing (28). Wellness from an Afrocentric perspective is seen as being ‘strong’, physically and spiritually (29), and is influenced by African spirituality, and family and community wellness (30, 31). From a Latin American perspective, health and wellness can be influenced by personal, familial, and collective wellbeing, a sense of belonging, and spirituality (32). A wellness approach also considers the impacts of sociopolitical, economic, and historical forces from people’s countries of birth or ancestral origins on their health and wellness.

Palliative care and patient navigation provide valuable supports for people with advanced cancer as well as those experiencing cancer disparities. Palliative care promotes the relief of suffering associated with a life-limiting illness and increased quality of life (33, 34), and advances social justice in the care of these populations (35, 36). Little is known about palliative care approaches with people of African and Latin American descent in Canada. African Canadians in Nova Scotia reported a lack of awareness of palliative care services (37), expressed a preference for care in the home with family and community participation (37, 38), as well as avoidance of health care institutions (38). Patient navigation may serve to address inequities affecting racialized groups and patients with advanced cancer. Patient navigation fosters empowerment and patient wellbeing, improves access to care (39), patient experience (40, 41), and reduces cancer and palliative care disparities (42–48).

## 2 Study aims

This research aims to work with racialized patients with advanced cancer of African and Latin American descent, their families, and communities to co-create a patient-oriented pathway to improve equity and wellness in cancer care. Our study aims are as follows:

1. Understand the lived experiences of equity and wellness of racialized patients with advanced cancer of African and Latin American descent;
2. Co-develop a patient-oriented pathway in cancer care to improve equity and wellness in patients with advanced cancer of African and Latin American descent; and
3. Explore the acceptability of the pathway with patients, families, community members, and health care providers.

For the purposes of this study, people of African and Latin American descent refer to any individual who self-identifies as belonging to the Black or Latin American communities respectively, in Canada, by birth or ancestry. The term Black is used interchangeably with people of African descent. We recognize language diversity when referring to people of African ancestry. People of Latin American descent may also identify as Black and there is wide heterogeneity within these racialized groups.

Community members are people from the Black or Latin American communities with either lived experience of cancer (e.g., patients, cancer survivors, family, significant others); or with an interest in improving equity and wellness in the care of patients with advanced cancer. According to the Canadian Cancer Society, racialized individuals “have racial meanings attributed to them as a group in ways that negatively affect their social, political and economic lives.”^49 p. 68^ People who are exposed to racialization processes experience racism and discrimination at a systemic level in the course of their lives (50). Although understudied, racism is gaining recognition as a public health threat, as it has been linked to discriminatory policies and environmental exposures (51).

## 3 Methods/Design

Following principles of health equity, intersectionality, and community wellness, we are undertaking a participatory research study. Intersectionality is a form of critical inquiry, which incorporates research traditions of social action and academic scholarship (52). Intersectional theory explores how social dimensions related to race, class, gender, sexuality and nationality, to name a few, interact to aggravate inequities (53–57). Community-based participatory research (CBPR) principles inform our participatory research approach. Building on social justice values and Paulo Freire’s critical theory work with people experiencing marginalization (58–60), CBPR contributes to the transformation and empowerment of those engaged in this form of research (61). In cancer care research, CBPR has been used to address inequities and promote patient engagement (62–65).

In following a patient-oriented approach, we have engaged patient partners from Latin American and African communities. Patient partners refer to individuals with lived experience of illness, caregivers, or friends (66). A study reported that engaging patient partners in cancer care contributed to improve wellness (67). We adhere to patient engagement principles, that is, inclusiveness, support, mutual respect, and co-building (66). These principles have guided the formation of a Patient Advisory Council (PAC), which is a diverse group of patient partners (AG, NR, FA, TW, MS, MCG, LZC, NMM) and community collaborators (BO, YO) who are involved in developing and informing this work. For example, patient partners have been involved in co-developing interview guides and recruitment materials. Members of our PAC are compensated for their time and contribution. The PAC oversees two conceptually linked research studies involving people of African and Latin American descent with advanced cancer including the study reported here and another one underway to increase access to palliative care (68). These studies have different aims and outcomes and have independent research funding. We have collaborated with the Alberta Strategy for Patient-Oriented Research (AbSPORU) Patient Engagement Team to enhance our patient-oriented research approach. They have provided research training to patient partners and the study team and will support our patient engagement efforts throughout the life of the study. We have also engaged the African Cancer Support Group in Alberta as a community partner and are in the process of approaching key community organizations.

### 3.1 Setting and sample

This research will be undertaken in Edmonton, Alberta and two sites in the Greater Toronto area in Ontario (Toronto and Durham). According to the 2021 Census of Population by Statistics Canada, the Latin American population in Canada continues to grow, with approximately 19,455 residing in Edmonton, 8,105 in Durham, and 92,455 in Toronto (69). Ontario reportedly has the largest Black population in Canada, however between 1996 and 2016 Alberta had the fastest growing Black population (70). The Black population in Canada largely resides in census metropolitan areas (71), with approximately 75,525 residing in Edmonton, 66,035 residing in Durham, and 265,005 in Toronto (69). At the Edmonton site, we are working with three teams at the Cross Cancer Institute (CCI) including Symptom Control and Palliative Care, Palliative Radiation Oncology, and Supportive Care. As a tertiary cancer center, the CCI provides cancer care for diverse populations (urban, rural, and remote) from the northern half of the province and some corridors of the northern territories and western provinces. In Toronto and Durham, there are two participating sites, including the Palliative Care Department at the Princess Margaret Cancer Centre and Lakeridge Health Region. By engaging three sites we will achieve our sample size, increase the diversity of our sample, and explore the perspectives of people in large Canadian urban areas.

We will aim for a total of 125 participants and will employ purposive sampling, that is, we will invite people who meet the following inclusion criteria: Patients with advanced cancer, their family members, and community members who self-identify as being of African or Latin American descent; and health care providers of African or Latin American descent, or those who have worked with the study populations. Caregivers or spouses who are not of African or Latin American descent will also be eligible to participate. We have determined the sample size considering our study aims as well as the diversity of settings and populations. Since we are interested in learning from the experiences of two racialized communities and their acceptability of the proposed patient-oriented pathway, we anticipate this sample size will be sufficient to achieve these goals (72). For study aim 1, we plan to accrue 10 patient-family dyads and 10 community members per participating site (30 participants per site). For study aim 3, we plan to accrue 10 patient-family dyads and 15 health care providers and community members (35 in total). This sample size will allow us to gather comprehensive data from participants in two Canadian provinces, generate an in-depth understanding of their lived experiences that reflects the diversity of research participants, and confidently determine the acceptability of the proposed patient-oriented pathway (study aim 3). Participant accrual will end when data collected are deemed sufficient to achieve study aims and now new themes are identified in data from new participants.

### 3.2 Participant recruitment strategy

We will recruit patient and family participants through our study sites. Eligible participants will receive a letter of initial contact from an intermediary at the clinical site and if interested, will be asked to provide verbal consent for the release of personal information to the study team. Alternatively, patients and their family members will also have the option to contact the study team directly via phone, email, or via a secure REDCap^©^ application accessed directly through a QR code on study posters or postcards. We will then contact them to explain the study and obtain consent (written or virtual/telephone). Consent to participate in the study may be written, explicit oral or implied depending on whether it takes place either in-person, via telephone or videoconference. Family members will be invited only with patient’s consent. All participants will provide consent in English. We will only consent participants not fluent in English when able to engage certified interpreters. The consent form will not be translated into other languages and the certified interpreter will read the statements in the participant’s preferred language.

To recruit patients, families, and community members, we will also use word of mouth through study participants, patient partners (members of the PAC), and the African Cancer Support Group, a community organization in Alberta dedicated to supporting African Canadians living with cancer; and other community organizations interested in collaborating with the study team. They will be able to support recruitment efforts if needed. In order to boost our recruitment efforts, we will offer a CAD $25 recruitment incentive to community members who refer participants to the study. We will distribute a letter of initial contact for patients, families, and community members through these same channels. In addition, research team members including study investigators will approach potential participants when possible, explain the study aim and invite them to participate. This may happen in places frequently attended by members of our populations of interest such as churches, grocery stores, or hair salons, among others. We may also do this during community events or other gatherings where we present the study to the community. Co-investigators who have clinical consults may share study materials with potential patient participants and invite them to participate.

We will post recruitment posters at the clinical sites, and distribute these as well to patient partners and interested community organizations. Patient partners, community organizations, and study team members may share the study poster on social media (e.g. Instagram, Facebook, WhatsApp, and LinkedIn). We will also place paper posters in the waiting room of our study sites and request digital display access on the sites’ public TV screens. In addition, we will distribute print versions of study postcards at our participating sites and through our community collaborators to be shared with potentially eligible participants. We will also employ posters and postcards in Spanish, Tigrinya, and Amharic. Poster and postcards may be translated into other languages in the future.

For the accrual of providers, we will ask the managers of selected clinical teams to circulate an informational email along with the letter of initial contact to their teams. Providers interested in participating in the study will contact the study team via email and we will then begin the consent process. The accrual of providers will take place under study aim #3 and we will request ethics approval for research activities associated with this aim after the patient-oriented pathway is developed.

### 3.3 Data collection methods

To accomplish aim 1, we will complete in-depth interviews with approximately 10 patient-family dyads in each site (30 dyads in total), or 60 patient and family members. We will undertake focus groups with 30 community members (approximately 10 in each site). Study aim 2 will involve the PAC formed by patient partners, community collaborators, and researchers. Other stakeholders may be invited to collaborate with study aim 2. To accomplish aim 3 of our study and explore the acceptability of the patient-oriented health equity pathway in cancer care, we will engage 35 participants including 10 patient and family dyads (20 in total), and 15 health care providers and community members. We will aim for similar numbers for participants of African or Latin American descent in our sample. We will provide an honorarium of $35 to each patient participant and a $25 gift card to health care providers as appreciation for their contributions.

All participants will be invited to complete an anonymous online sociodemographic questionnaire via a secure REDCap^©^ online form including email, name, telephone number, age, gender, race and ethnicity, household income, employment status, marital status, education, country of birth, first language and other languages spoken, place of residence, family support in Canada, number of children and religion. During the interview, we will collect basic clinical data from patients such as cancer diagnosis, stage, time since diagnosis, and treatments. This information will be voluntary and based on participants’ self-report. Providers will be asked about their years of experience as a health professional, specialization, years in oncology or palliative care (if applicable), and full-time equivalent status. Community members and family members will be asked to complete the sociodemographic questionnaire only. No health information will be collected from family members or community members. Except for health care professionals, all participants will be asked if they are interested in participating in other studies looking for people of similar characteristics. If they are, we will mention study opportunities currently led by the study principal investigator.

Upon completion of interviews and focus group discussions, data will be uploaded to a secure online platform, transferred to the principal investigator’s research directory, and sent for transcription via a secure server. The transcription company will fully anonymize transcripts by removing any identifying information. Transcripts will be verbatim and verified by a research assistant for accuracy. Research data will be securely stored in the principal investigator’s home institution.

In line with our participatory approach, we have discussed the research plan with the PAC since the planning stages and we will continue to adjust as needed based on members’ feedback. Below, we discuss research activities in relation to our specific study aims.

#### 3.3.1 Study aim 1: Understand patients’ lived experiences of equity and wellness

This aim will explore participants’ meanings of equity and wellness based on their own experiences. We will also examine their views on how palliative care and patient navigation can enhance equity and wellness. This knowledge is important to ensure a patient-oriented approach in the design of the pathway. We will conduct in-depth interviews with patient-family dyads. This interviewing approach considers a relational understanding of experience where both patients and their family members share distinct yet interrelated understandings and interpretations of their experiences. The interviews will be 60 minutes in duration and we will conduct them over the phone, via videoconferencing, or in person, at a time and place that is convenient to participants. We have finalized our patient and family interview guide with PAC members (see **Supplementary File 1**).

We will also undertake focus groups to explore the views of community members of African or Latin American descent concerning equity and wellness in the context of people living with advanced cancer. The focus groups will also serve to explore their views on palliative care, and how palliative care and patient navigation can enhance equity and wellness. We will complete 4 two-hour focus groups via videoconference with about 8 community members each. The focus group discussion guide has been finalized with feedback from the PAC to ensure relevance and cultural appropriateness (see **Supplementary File 2**).

Interviews and focus group discussions will be digitally recorded. Although we will conduct interviews and focus groups in English, we will explore opportunities to conduct them in participants’ home language, if preferable by participants. Through our community collaborations, we may be able to engage the services of certified interpreters to assist with data collection in other languages. Additionally, several of our patient partners bring rich linguistic diversity as well as qualitative research training and may be able to facilitate interviews or focus groups in other languages. Interviews and focus group guides will be in English and facilitators conducting the interview or focus group in other languages will ask the questions in the participant’s preferred language. Interviews conducted in other languages will be first transcribed in the original language when possible and then translated into English by a certified interpreter. The translations will be then verified by a second certified interpreter. In order to ensure cultural appropriateness during interviews or focus groups, we will engage one or two patient partners as co-facilitators.

#### 3.3.2 Study aim 2: Develop a patient-oriented pathway to improve equity and wellness

Based on research findings from the interviews and focus groups, we will then create a patient-oriented pathway to improve equity and wellness in patients with advanced cancer. Informed by patient-oriented research (66), a patient-oriented pathway is a set of actions developed with patient partners, based on patient-identified priorities, and aimed at improving outcomes, in this case, equity and wellness in patients with advanced cancer of African or Latin American descent. In the development of the pathway, we will build on research findings from study aim 1, and will follow the guidance of patient partners and community collaborators. We will explore how palliative care, patient navigation, and community wellness may inform this pathway. The PAC will meet every month for 2 hours throughout the study and will review documents prior to meetings. The work on the pathway will begin soon after preliminary findings are available. Freire’s cultural circles approach will guide the development of the pathway (58, 73, 74). Cultural circles are a liberating practice where participants bring their own knowledge and lived experiences and engage in work of conscientization and problematization of conditions of oppression or marginalization (58, 73–75), in this case, inequities in cancer care. The cultural circle will meet on a regular basis to examine through continuous critical-reflexive dialogue the underlying roots of these conditions and explore ways of addressing them. Cultural circle members identify and develop key themes exposing conditions of oppression or marginalization and actions needed for liberation (58). These themes will inform the development of the components of the patient-oriented pathway. In our work with the PAC, we have held critical discussions of issues surrounding cancer care inequities. These conversations will provide the basis for the cultural circles and will guide the ensuing development of the pathway.

Guiding questions will include: ‘Based on patient experiences and recommendations, what would a patient-oriented pathway look like?’ ‘Is there a place for palliative care, patient navigation, and community wellness in this pathway?’ ‘How can the pathway improve accessibility and inclusivity in cancer care?’ ‘In what way would this pathway respond to patient priorities and concerns?’ The principal investigator and study coordinator will facilitate these meetings, and a research assistant will help with note taking. Study investigators (AS, SW, AyS, FH, JAC, HO, PCP, BS, MS, OAR) and implementers/decision makers (SW, AyS) will take part when necessary to provide input concerning feasibility, requirements, and challenges from a health system perspective. All study team members will have an opportunity to provide feedback on the pathway.

#### 3.3.3 Study aim 3: Explore the acceptability of the pathway

The pathway will be documented in writing and will include a title, lay and professional summaries, a description statement, background and rationale, goals, components and activities, and risks and benefits. Acceptability refers to the degree to which a service may be deemed desirable by potential users (76, 77). This is a vital step to ensure the pathway reflects patient priorities. The purpose of exploring the acceptability will be to allow for adjustments to the pathway and remedy potential issues, biases, and assumptions. We will use qualitative methods to explore acceptability (78), with a sample of 10 patients and family dyads (20 participants), and 15 health care providers, and community members from any of the study sites. From a qualitative perspective, this sample will assist us to gather enough data to examine acceptability (79). We will invite participants to a semi-structured 30-60 minute interview. We will share the proposed pathway and will ask open-ended questions about pathway appropriateness, effectiveness, risks, and convenience (76). Interviews will be recorded and transcribed verbatim. We will create summaries of data highlighting key findings and revise the pathway based on what we learn from research participants.

Following adjustments, we will organize 6 conversation cafés (4 in person and 2 virtual) to disseminate the pathway with members of Latin American and African communities in Edmonton and the Greater Toronto Area to learn about their perspectives on the pathway and possible implementation barriers and facilitators. The cafés will last 1-2 hours and will be an informal gathering in the community or virtually. These cafés will inform the pathway implementation plan and a future pilot research study to explore feasibility and effectiveness. We will hold one virtual café in each site with health care stakeholders (front line providers, managers, program leaders, and decision makers) to hear their views on the pathway and implementation barriers and facilitators. Conversation cafés will be a knowledge dissemination activity and attendees will not be research participants. We will plan to have 25 attendees per café.

### 3.4 Data analysis

We will utilize thematic analysis (80) and will manually analyze the data. Transcripts will first be read as a whole by two independent research team members. The process of manual analysis requires line by line reading to identify key words, phrases, and revealing passages in the data. The analysis follows an interpretive approach (80, 81), through which we will pay close attention to stories shared by participants. Each team member will generate preliminary themes and sub-themes based on their reading of the transcript. Next, team members will meet to examine their emerging themes and sub-themes, and their corresponding transcript excerpts, and achieve consensus regarding these. Differences will be resolved by consensus together with the lead researcher or a senior team member. A theme will correspond to a unique aspect of participants’ experiences of equity and wellness in cancer care; and together, themes will yield an in-depth picture of their experiences of equity and wellness.

In the course of data analysis, emerging themes will be examined in light of new data and will then be amalgamated with other themes, removed, or fully described until we achieve a thoughtful understanding of experience. Although we will seek to generate a comprehensive picture, this rendering is never complete because of the large diversity of human experiences, contexts, and people. We will follow preliminary themes and sub-themes in the analysis of subsequent interviews or focus groups, however, the thematic structure will be evolving and finalized once data collection is complete. To promote consistency across themes and sub-themes, a team member with expertise in thematic analysis will be involved in data analysis, we will keep a list of definitions of themes, hold regular check ins with the lead investigator, and allocate sufficient time to discuss differing interpretations. In addition, to increase trustworthiness of findings, we may conduct follow up interviews with selected participants and will discuss themes and sub-themes with patient partners.

In line with an intersectional approach rooted in critical theory (58), we will examine how social dimensions contribute to participants’ lived experiences of equity and wellness. Inequities in cancer care often result from the impacts of interacting social dimensions. Principles of health equity and intersectionality will inform an analysis that takes into account the concurrent impacts of gender, race and racism, socioeconomic status, being a member of a racialized community, and language barriers, among others. The impact of gender will be integrated into data analysis. While we do not have an explicit focus on gender diverse identities, people who self-identify as belonging to gender diverse communities may take part in the study. We will be attentive of the interplay of gender identity and participants’ experiences of equity and wellness and facilitate representation of diverse genders in data analysis when possible. Understanding the influences of gender and related social dimensions on participants’ experiences will help us clarify how equity and wellness manifest in their cancer journey, and examine possible mechanisms underlying inequitable care outcomes.

### 3.5 Ethical considerations

This study received ethics approval from the Health Research Ethics Board of Alberta Cancer Committee on May 17, 2023 (HREBA.CC-22-0373). Informed consent will be obtained from participants prior to data collection. We will strictly adhere to Canada’s Tri-Council Policy Statement (TCPS2) on the conduct of research with human beings (82). We will also enact the TCPS2 core elements to ensure the ethical integrity of this study including respect for persons, concern for welfare, and justice (82). Study activities pose minimal risk to study participants, that is, the risks are not greater than those encountered in daily life. Although the study may yield no benefits to study participants, we hope this study will make a significant contribution to the scholarly community and also to the clinical care of our study populations. We will be pay close attention to ethical considerations related to qualitative research such as the ongoing consent process, privacy and confidentiality, data collection practices, research partnership building, data ownership, and limitations concerning the generalizability of research findings, among others (82).

We will protect participants’ anonymity and confidentiality by removing any identifying information from transcripts and storing files in a secure health research data repository (HRDR). The HRDR provides access to data through a secure virtual private network that offers a remote and encrypted connection. Only authorized and authenticated users can access HRDR study folders. The transcription company will have temporary access to the recordings. All study team members and the transcription company will sign a confidentiality agreement. In addition, data will not be made publicly available in order to protect participant data as well as honor community partnerships and project partners’ preferences regarding public access to data. We will ensure a safe and culturally respectful strategy to data accessibility and ownership that is developed in consultation with the PAC.

During data collection procedures, we will be attentive to any signs of fatigue or emotional distress and pause or stop the interview or focus group if necessary. We have checked the interview and focus group guides for cultural fit and to ensure questions reflect tactfulness and thoughtfulness. We will recruit patients who are clinically stable and ensure our data collection procedures do not become burdensome to participants at any point. Since we follow a participatory approach, modifications to the protocol will be submitted to the ethics board whenever revisions are made together with our PAC. Data will be stored for at least five years following institutional policies or until the end of scholarly activity related to this study.

## 4 Discussion

This study will make an original contribution to the fields of health equity and oncology. A significant feature of this research is a focus on two largely underrepresented populations in health research in Canada including people with advanced cancer and members of racialized populations. These underserved populations face diverse gaps and barriers in access to cancer care and related health care resources and services (49). Engagement with people with advanced cancer as well as racialized communities requires careful consideration of their health status, timing and strategies, recognition of systemic inequalities, the impact of racism, and their sociocultural identities (49).

This study will increase knowledge of how Black and Latin American people with advanced cancer navigate inequitable health and social systems in their life course. We hope to build trust through patient and community engagement, learn from the communities themselves, and contribute to the development of culturally safe and respectful research environments. The study will generate evidence of cancer care inequities in Canada affecting racialized communities that is rooted in the lived experiences of members of these communities. Understanding these inequities is a necessary step to design programs directed at improving inequitable cancer care outcomes. This research will contribute to address an identified need to advance health equity research to improve access to cancer care for underserved populations in Canada (83). Although health equity progress in Canada has been described as slow (84), the positive response to this research among diverse stakeholders reflects a growing concern and interest in generating actionable change. This research will advance health equity in cancer care by envisioning together with affected communities real-world solutions to tackle health disparities.

The main study outcome will be an increased understanding of how we can improve equity and wellness in Latin American and Black peoples with advanced cancer. As well, we will gain knowledge of the relevance of a patient-oriented approach when doing health equity research with Latin American and Black peoples in Canada. We anticipate the patient-oriented pathway will cover a range of domains including actions directed at patients, families, and communities, actions addressing health care practices, as well as system-level actions targeting racial inequities. The pathway will likely integrate an intersectional approach, which has shown promising potential when caring for individuals with cancer and non-cancer diagnoses experiencing socioeconomic inequities (85). In this study, we will gain an understanding of the acceptability of the pathway. We hope to formulate new research projects with project partners to operationalize and evaluate the pathway in real-world health care settings.

### 4.1 Alternative approaches and barriers to implementing the study protocol

Our research team brings diverse strengths to this research project. We have an established research record in cancer care, health equity, and patient oriented-research as well as knowledge of Latin American and Black peoples. Our patient partners and community collaborators not only bring expert knowledge and lived experiences as members of racialized communities, but also interest in advancing equity in the care of our study populations. We may experience challenges implementing this study protocol due to lack of trust and interest, limited community engagement, and difficulties with participant accrual. Patient and community engagement are vital to building trusting relationships with communities. We have engaged patient partners from the planning stages of this research project to ensure this study responds to community priorities. Our PAC oversees the research project and offers cultural guidance so that our research processes are culturally safe. We will foster reciprocal and respectful relations with project partners to maintain and strengthen community engagement. Through collaboration and shared leadership, key features of CBPR (86), we will foster inclusive research practices. We will also evaluate our patient and community engagement processes and modify them as needed. In order to achieve our projected sample size, we will enact an array of participant accrual strategies and continue to explore alternatives. We have engaged members of our communities of interest to support participant recruitment, provide participants a small honorarium as a gesture of appreciation, and collaborate with community-based organizations. These actions are identified as facilitators of recruitment in CBPR studies (87). We may approach new community collaborators, organizations serving our study populations and potentially other clinical sites to increase opportunities for recruitment.

### 4.2 Knowledge dissemination

The main study output is the patient-oriented pathway in cancer care to improve equity and wellness in racialized populations of African and Latin American descent. Additional study outputs include open-access publications, conference presentations, and innovative dissemination products to highlight research findings. We will disseminate findings and the patient-oriented pathway with project partners including cancer care and health system leaders, oncology and palliative care teams, organizations serving racialized communities, and community members. The engagement of the PAC throughout the research will facilitate an ongoing knowledge exchange process with our project partners. This will contribute to the production of culturally relevant research findings and evidence, and will facilitate the dissemination of knowledge to the communities. We will work with project decision makers/implementers and health system partners to co-develop strategies to disseminate the pathway with relevant health system decision makers and policy makers. We will co-design innovative digital products to disseminate research findings and the proposed pathway including video clips and infographics.

The potential for scaling the patient-oriented pathway to other racialized communities or healthcare settings exists. This research will generate opportunities for new collaborations and expanded community engagement with racialized and other underserved communities not directly involved in this research. We will explore ways of engaging these communities to learn from their perspectives concerning the potential resonance of the patient-oriented pathway with their communities. This work can inform future research to apply and expand our research findings in the context of diverse communities. We will be mindful of the great diversity within communities and make a concerted effort to recognize people’s own identities within their own collectives.

### 4.3 Limitations

African and Latin American peoples comprise highly heterogeneous communities. One of the study limitations is that we will not be able to honor the richly diverse character of African and Latin American peoples in the study. We will consider this limitation in our interpretations and study outputs to avoid misleading or stereotyping conclusions. This is a community-based qualitative participatory study and while research findings will be specific to the context and the populations of this study, we hope they can illuminate clinical practice with similar populations and inform the design of research projects to continue to advance knowledge in this area. This research project will provide insights and understandings that can prompt critical reflection and the development of new health equity research paths. There is also potential for research findings to inform practice with other racialized and underserved populations experiencing cancer care inequities.

## 5 Closing remarks

This study emphasizes a health equity focus that brings attention to systemic, unfair, and avoidable disparities in cancer care affecting Latin American and Black peoples in Canada. A significant highlight of this study is the patient and community engagement in the design and conduct of the study to ensure the study honors the worldviews and traditions of Latin American and Black communities. The study advances health equity research in Canada and increases access to health research to underrepresented populations within the Canadian cancer research ecosystem. There is potential for the patient-oriented pathway to be scaled to other racialized and underserved communities. Lastly, through intersectoral and interdisciplinary collaboration, we hope to contribute to the building of an equitable Canadian cancer care system.
