# Supplementary material for: Improving equity and wellness in cancer care with people of Latin American and African Descent: a study protocol
Source: Front Oncol. 2025 Feb 26;15:1469037. doi: 10.3389/fonc.2025.1469037 (PMC11896859; doi:10.3389/fonc.2025.1469037)
Supplement: Supplementary file 1 [file DataSheet1.pdf]

Supplementary Material

Interview Guide for Patients and Family Members

1. Tell us a little bit about yourself and your illness experience

Probes

- What can you tell us about your experiences since the time you began feeling unwell?
- What would you like to share about your illness experience?
- Are there experiences that come to mind during this time?

2. What can you tell us about your ability to live well during this time?

Probes

- What does living well mean to you? Living well could include things like emotional, physical, psychological, financial, or spiritual well being
- What things have helped you during this time to maintain or improve your ability to live well during this time?
- In your view, what can be done or what needs to change in health care to support your ability to live well with cancer

3. Can you describe your experiences accessing care since you started feeling sick?

Probes

- Have you experienced issues related to income, housing, employment, social support, transportation, childcare, family demands, or other living and working conditions? Would you like to tell us more?
- Have you experienced issues as a result of your gender, race, language, disability, or other social identities? What can you tell us about this?
- Have you experienced racism or discrimination? Can you tell us more about this?

4. What do you know about palliative care?

Note for the interviewer: *Palliative care is a health service for people who are living with a life-limiting illness. The goal of palliative care is to support the relief of symptoms and promote quality of life for both patients and their families.*

Probes

- Are you familiar with the term ‘palliative care’? What does it mean to you?
- Would you be interested in learning more about palliative care?
- Have you been able to access these services in the hospital or the community?
- Do you have questions or concerns you would like to discuss about palliative care?
- Do you believe palliative care could contribute to your personal, familial, and community wellbeing?

5. **What do you know about patient navigation?**

Note for the interviewer: **In Alberta, there is a patient navigation program where registered nurses with specialized training in cancer care help people understand treatment choices and test results, and assist with the physical, practical and emotional challenges that come with a cancer diagnosis. Please note patient navigation is only offered to specific patients in Alberta- Indigenous, adolescents/young adults, and rural Albertans.*

Probes

- Are you familiar with the term 'patient navigation'?
- Have you had an experience with a patient navigator?
- Would you be interested in accessing this service if it was available?
- What things would you expect from a patient navigator?

6. **Suggestions for improvement.**
   - Participant(s) will be invited to share suggestions to help with their equity and wellness experiences
   - Were your expectations different from your cancer care experience?
   - What can be done to remove the barriers in yours or other people's cancer care journeys?
   - Would you like to share something that we haven't discussed?

The following questions apply to patient participants only

1. What is your diagnosis? Have you been diagnosed with other health conditions?
2. When were you diagnosed with cancer for the first time?
3. When were you diagnosed with cancer?
4. Which of the following cancer treatments have you received? Please indicate dates if you can recall
   a. Radiation
   b. Chemotherapy
   c. Surgery
   d. Immunotherapy
   e. Other (describe)
5. Are you currently receiving palliative care?
6. Which of the following palliative care services are you receiving? (if participant is receiving palliative care)
   a. Hospital outpatient palliative care team
   b. Palliative home care
   c. Family physician services
   d. Hospice
   e. Other (describe)
7. Will you be willing to attend any community events where we share study findings?
