# Supplementary material for: Improving equity and wellness in cancer care with people of Latin American and African Descent: a study protocol
Source: Front Oncol. 2025 Feb 26;15:1469037. doi: 10.3389/fonc.2025.1469037 (PMC11896859; doi:10.3389/fonc.2025.1469037)
Supplement: Supplementary file 2 [file DataSheet2.pdf]

frontiers

Supplementary Material

Focus Group Guide for Community Members

1. **Tell us a little bit about yourself and your experience with cancer or palliative care**

Probes

- Please share in what capacity you have had experiences in this area (e.g. neighbour, community member, faith-based role, care provider, policy maker, family member, etc)

2. **What can you tell us about people's ability to live well when living with cancer?**

Probes

- What does living well mean to you? Living well could include things like emotional, physical, psychological, financial, or spiritual well being
- What things would help people in your community living with cancer and their families to live well during this time?
- In your view, what can be done in health care to support their ability to live well?

3. **In your view, what is it like to access cancer care for people in your community?**

Probes

- Are there issues related to income, housing, employment, social support, transportation, childcare, family demands, or other living and working conditions? Would you like to tell us more?
- Do they experience issues as a result of gender, race, language, disability, or other social identities? What can you tell us about this?
- In your opinion, are some of the experiences related to racism or discrimination? Can you tell us more about this?

4. **What do you know about palliative care?**

Probes

- Are you familiar with the term 'palliative care'? What does it mean to you?
- Would you be interested in learning more about palliative care?
- Are people with cancer in your community able to access these services in the hospital or the community?
- Do you have questions or concerns you would like to discuss about palliative care?
- Do you believe palliative care could contribute to the personal, familial, and community wellbeing of people with cancer who are part of your community?

5. **What do you know about patient navigation?**

Probes

- Are you familiar with the term 'patient navigation'?
- Would people in your community living with cancer be interested in accessing this service if it was available?

- What things would you expect from a patient navigator?

6. Suggestions for improvement.

Probes

- Participant(s) will be invited to share suggestions to help with the equity and wellness experiences of patients with cancer and their families
- What could be done to remove the barriers we discussed in cancer care journeys?
- Would you like to share something that we haven’t discussed?

7. Will you be willing to attend any community events where we share study findings?
